# Effects of High-Resolution Natural Sound with Inaudible High-Frequency Components on Healing, Symptoms, and Sleep Satisfaction in Terminally Ill Cancer Patients

**DOI:** 10.1089/pmr.2024.0089

**Published:** 2025-02-07

**Authors:** Yasutaka Shimotsuura, Keisuke Ishizuka, Takashi Kawaguchi, Kazue Umetsu, Mariko Harada, Yujiro Inoue, Emi Kubo, Kazuhiro Kosugi, Takashi Igarashi, Seiya Enomoto, Hidehiko Taniyama, Tetsuo Iwata, Takuhiro Yamaguchi, Yoshihisa Matsumoto, Tomofumi Miura

**Affiliations:** ^1^ Department of Palliative Medicine, National Cancer Center Hospital East, Kashiwa, Japan.; ^2^ Department of Clinical Assessment, Tokyo University of Pharmacy and Life Sciences, Hachioji, Japan.; ^3^ Department of Pharmacy, National Cancer Center Hospital East, Kashiwa, Japan.; ^4^ JVCKENWOOD Corporation, Yokohama, Japan.; ^5^ Division of Biostatistics, Tohoku University Graduate School of Medicine, Sendai, Japan.; ^6^ Division of Biomarker Discovery, Exploratory Oncology Research and Clinical Trial Center, National Cancer Center Hospital East, Kashiwa, Japan.

**Keywords:** cancer, healing, high-resolution natural music with inaudible high-frequency components, music, palliative care

## Abstract

**Objectives:**

This study aimed to assess the effects of high-resolution natural sound with inaudible high-frequency components (HNIH) on healing, symptoms, sleep satisfaction, and autonomic nerve function among terminally ill cancer patients.

**Methods:**

We conducted a single-arm, open-label study of 4-hour HNIH for 20 terminally ill cancer patients. We evaluated the healing state, symptoms (Japanese version of the Edmonton Symptom Assessment System-Revised, ESAS-r-J), global impression, and heart rate variability at 30 minutes (T2) and 4 hours (T3) after starting HNIH and sleep satisfaction the next morning (T4).

**Results:**

A total of 18 participants were evaluated (mean age: 69.4 years; 33.3% female). Post-intervention, there was a nonsignificant increase in Healing Scale scores at T2 (mean difference: 5.3, 95% confidence interval [CI]: −1.2 to 11.8, *p* = 0.106), but a significant increase at T3 (mean difference: 6.6, 95% CI: 1.0 to 12.3, *p* = 0.024). Specific ESAS-r-J scores demonstrated significant improvements in anxiety (mean difference at T2: −1.2, 95% CI: −1.99 to −0.34, *p* = 0.008; T3: −1.2, 95% CI: −1.99 to −0.34, *p* = 0.008), tiredness (mean difference at T2: −0.6, 95% CI: −1.18 to −0.04, *p* = 0.037), and shortness of breath (mean difference at T2: −1.0, 95% CI: −1.72 to −0.28, *p* = 0.010). Moreover, 66.7% of participants reported improved general conditions at T2 and T3, whereas 50% reported enhanced sleep satisfaction at T4. Heart rate variability analysis revealed a decreased low-frequency/high-frequency ratio in 55.6% of participants at T2 and 44.4% at T3.

**Conclusions:**

The present single-arm study showed that HNIH potentially enhanced healing, alleviated symptoms such as anxiety, tiredness, and shortness of breath, and improved sleep satisfaction in terminally ill cancer patients.

## Background

As the prevalence of advanced cancer increases globally, there is a growing need for palliative care.^1^ Palliative care aims to relieve irritable symptoms and distress and improve the quality of life (QOL) for patients and their caregivers. For terminally ill cancer patients, nonpharmacological treatment is important to provide healing and relaxing effects with fewer side effects. Therefore, the development of effective nonpharmacological approaches is anticipated.^2^

Among these, auditory stimulation, particularly music therapy, has been shown to have beneficial effects on psychological symptoms such as anxiety and depression, as well as physical symptoms such as pain and fatigue.^3^ Among terminally ill cancer patients, music therapy reduced pain and improved QOL.^4^ These benefits have been seen both with music therapy interventions guided by trained music therapists, as well as prerecorded music without therapist guidance.^3^ In addition, previous research suggested that the therapeutic potential of natural sounds in improving psychological outcomes has led to its application in various clinical settings.^5^ Despite the accumulating evidence supporting the therapeutic use of sound, little is known about the potential benefits of high-resolution natural sounds with inaudible frequency components (>20 kHz). Although some studies have suggested physiological responses to inaudible frequency components in healthy persons, these effects are not yet fully understood among terminally ill cancer patients.^6–8^

Therefore, the purpose of this study was to investigate the impact of high-resolution natural sound with inaudible high-frequency components (HNIH) on healing, symptoms, the general condition, sleeping, and autonomic nerve function in terminally ill cancer patients.

## Materials and Methods

This was a single-center, single-arm open-label study to explore changes in the healing state, symptoms, autonomic functions, and sleep satisfaction using HNIH. This study was performed according to the Declaration of Helsinki, approved by the National Cancer Center Institutional Review Board (No. 2021-227), and registered in the Japan Registry of Clinical Trials (jRCT1032210523, https://jrct.niph.go.jp/en-latest-detail/jRCT1032210523). All participants provided written informed consent. We enrolled participants and followed up between December 27 2021 and June 30 2022. Regarding data sharing, in accordance with the ethical guidelines for medical and biological research involving human subjects in Japan,^9^ our protocol did not allow free data sharing without additional approval by the National Cancer Center Institutional Review Board. We receive the request of data sharing from pal-data-request@east.ncc.go.jp. The secretariat of this e-mail is a member of local data administration committee of the National Cancer Center Hospital East and responsible for ensuring data access.

### Participants

We enrolled cancer patients as follows: (1) who were ≥20 years old, (2) who were admitted to the palliative care unit at the National Cancer Center Hospital East, (3) who could answer the questionnaires in Japanese, (4) whose Karnofsky Performance Status (KPS) ranged from 50 to 100, and (5) who did not meet criteria for confusion/delirium based on confusion assessment methods. Patients were excluded if they had the following: (1) life prognosis estimated to be within 24 hours and (2) difficulty wearing a 24-hour electrocardiograph (ECG).

### Protocol

**FIG. 1.**
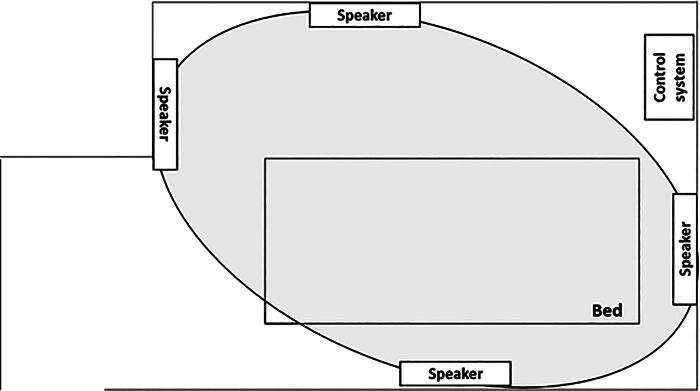
Sound system in hospital room. Four speakers of the high-resolution natural sound system were placed on the floor against the walls of the hospital room so that the bed would be surrounded by high-resolution natural sound (gray round).

**FIG. 2.**
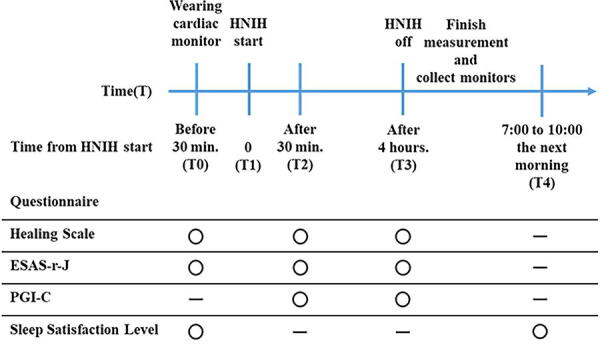
Study schema. HNIH, high-resolution natural sound with inaudible high-frequency components; ESAS-r-J, Japanese Version of the Edmonton Symptom Assessment System-Revised; PGI-C, patients’ global impression of change.

Participants were asked to wear a 24-hour ECG (RAC-5103, Nihon Kohden Corp., Tokyo, Japan) from the start of the measurement until 4 hours after the start of the high-resolution natural sound and to stay calm in their hospital beds in the hospital room. Four speakers of the high-resolution natural sound system (KooNe, JVCKENWOOD Corp., Yokohama, Japan) were placed on the floor against the walls of the hospital room so that the bed would be surrounded by high-resolution natural sound (sounds of the Suwa forest and the Togakushi river with quantization bit rate: 24 bit and sampling frequency: 96 kHz) (Fig. 1). At first, the measurement was performed for 30 minutes. Then, the sound was played at 40 dB for 4 hours, and the speakers and ECG were removed (Fig. 2). Participants were asked to stay calmly on their beds, and not to walk around, watch TV, and listen to other music during the sound intervention. Thereafter, participants resumed their normal activities. Time points were set at the start of the measurement (T0), at the start of the sound (T1), at 30 minutes (T2) and 4 hours (T3) after the start of the sound, and the next morning (T4). Medications were not changed during study periods. All procedures were performed by Y.S., K.I., and T.M. There was no payment made to the participants.

### Questionnaires

Healing state, symptoms, and patients’ global impression of change (PGI-C) were evaluated at T0, T2, and T3, whereas sleep satisfaction was assessed at T4, all using a self-reported questionnaire.

Healing state was evaluated using the Healing Scale, which was developed by the Nihon University College of Art to measure the psychological state and changes in a subject’s “healed” axis that arise from impressions of external objects, including art appreciation and the environment.^10^ The Healing Scale consists of 30 items using a 3-point Likert scale (0: disagree, 1: slightly agree, 2: agree). The total Healing Scale score, which was the sum of 30 items, was rated as: “not healed,” score of 0 to 14; “healed,” score of 15 to 31; “quite healed,” score of 32 to 48; and “extremely healed,” score of 49 to 60. The total Healing Scale score indicates the extent to which the subject art evokes healing feelings. The Healing Scale comprises six subscales, each representing a unique type of psychological healing experienced through art or other sources of comfort as follows: (1) Nagomi (Relieved Healing): This subscale reflects a state of calm and relaxation, where the individual feels a sense of relief, warmth, and reassurance. It embodies a peaceful and comforting emotional experience. (2) Kiwami (Self-Developed Healing): This subscale captures the inspiration and motivation derived from engaging with a work. It encourages feelings of hope, courage, and the drive to refine oneself, fostering personal growth and development. (3) Kiyoraka (Pure Healing): This subscale represents a serene and purified mental state. It evokes clarity and a sense of cleansing, often bringing about a sublime emotional experience that enhances a tranquil and noble mood. (4) Uruoi (Refreshing Healing): This subscale indicates a refreshing and revitalizing psychological state. It is associated with relaxation and renewal, offering a light-hearted, refreshed, and relaxed feeling. (5) Hazumi (Merry Healing): This subscale expresses joyfulness and lightness of heart. It involves a playful child-like delight that lifts the spirit, evoking a feeling of emotional buoyancy and cheerfulness. (6) Mushin (Selfless Healing): This subscale represents a state of emptiness and stillness, where the individual enjoys simply being present without active thought. This experience aligns with the concepts of void in Mahayana Buddhism or nonaction in Taoism, bringing about a peaceful content state of nothingness. The Healing Scale also has two more subscales as follows: “therapeutic energy,” as a force that comforts and heals the heart, and “self-help energy,” as a force for self-actualization, self-disclosure, and supremacy. The Healing Scale is only available as the Japanese version.

Symptoms were evaluated using the Japanese version of the Edmonton Symptom Assessment System-Revised (ESASr-J). ESASr-J consists of a 10-item symptom battery as follows: pain, tiredness, drowsiness, nausea, appetite loss, shortness of breath, depression, anxiety, well-being, and other symptom using an 11-point numerical rating scale.^11^

PGI-C was commonly used for patient-reported outcomes to evaluate changes in their conditions during study periods.^12^ PGI-C consists of a 7-point Likert scale from 1, very much improved, to 7, very much worse.

Sleep satisfaction was assessed using an original scale, which asked the following: “Were you satisfied with your sleep last night?” answered using a 6-point Likert scale from 0, strongly disagree, to 5, strongly agree.

### Heart rate variability

R-R intervals (RRIs) were analyzed by software (QP-550D, Nihon Kohden Corp., Tokyo, Japan). Frequency domain measures were calculated for 5-minute segments of RRI data using Kubios HRV Premium ver. 3.5.0 software. The frequency domain was analyzed by fast Fourier transform and included high frequency (HF, 0.15–0.4 Hz), low frequency (LF, 0.04–0.15 Hz), very low frequency (0.993–0.04 Hz), and the LF and HF ratio (LF/HF).^13^ HF, LF, and LF/HF reflect parasympathetic (vagal) activity, sympathetic activity, and overall balance between sympathetic and parasympathetic activities, respectively.^14^ These parameters were frequently adopted as markers of stress in various studies.^14–16^

### Adverse events

We collected data on only life-threatening severe adverse events as the safety of music therapy is well-known.^3^

### Statistical analysis

In this exploratory study, based on the results of prior research, assuming a significance level (α) of 0.05, an expected mean of 25, and a standard deviation (SD) of 17, when conducted with 20 participants, the 95% confidence interval (CI) for the mean of the Healing Scale is calculated to be between 17 and 33 points. This range is considered reasonable as it is judged to be “healed.” Therefore, we set the sample size as 20.

The primary endpoint was the mean score of the total Healing Scale score at T2. Descriptive statistics were used to summarize study results. Continuous variables are expressed as the mean with SD or median with an interquartile range. Changes of each score from T0 were analyzed using the paired *t* test. Estimated mean changes from T0, 95% CIs, and the effect size (ES) were also calculated by dividing the difference between the post-intervention mean and the preintervention mean by the pooled SD. The pooled SD is the square root of the sum of the squared SDs of the preintervention and post-intervention measurements, divided by 2. PGI-C was divided into two groups as follows: 1 to 3 as “improved group” and 4 to 7 as “no change to worsening group.” Sleep satisfaction levels were divided into two groups as follows: 0 to 2 as “unsatisfied group” and 3 to 5 as “satisfied group.” The change of sleep satisfaction was evaluated using comparison between T0 and T4 and divided into the following: “improved group” and “no change to worsening group.” LF/HF during T0 to T1 (T0-1), T1 to T2 (T1-2), and T2 to T3 (T2-3) was calculated, respectively. The change of LF/HF was calculated using LF/HF during T1-2 or T2-4 by LF/HF during T0-1 and divided into the following: “decreased group” or “no change to increasing group.” Statistical analysis was performed using JMP 14.0 (SAS Institute, Cary, NC, USA).

## Results

### Patients’ characteristics

**FIG. 3.**
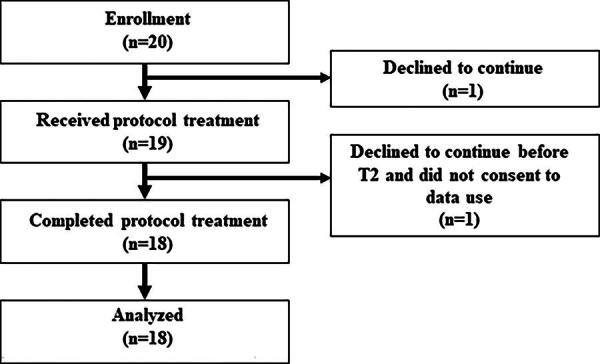
Patient flowchart.

Between November 2021 and June 2022, 20 patients were enrolled in the present study and 18 patients were analyzed (Fig. 3). Patients’ backgrounds are shown in Table 1. The mean age was 69.4, and females comprised 33.3%. A total of 83.4% of patients had KPS of 50 to 60.

**Table 1.** Patients’ Characteristics

Patients’ characteristics (*N* = 18)			
Variables	Category	*N*	%
Age	(Mean, SD)	69.4	7.5
Sex	Male	12	66.7
	Female	6	33.3
Cancer type	Urinary tract	3	16.7
	Lung	3	16.7
	Gallbladder and pancreas	3	16.7
	Colon	3	16.7
	Other	7	39.1
Metastasis	Liver	7	38.9
	Bone	7	38.9
	Lung	4	22.2
KPS	50	10	55.6
	60	5	27.8
	70	1	5.6
	80	1	5.6
	90	1	5.6

SD, standard deviation; KPS, Karnofsky Performance Status.

### The change of healing state

The mean total Healing Scale score was 20.9 (95% CI: 14.2–27.6) at T0, 26.2 (21.3–31.0) at T2, and 27.5 (20.8–34.2) at T3. Therefore, the healing state at both T2 and T3 was determined as healed. The proportions of participants categorized as “not healed,” “healed,” “quite healed,” and “extremely healed” were 27.8%, 61.1%, 11.1%, and 0% at T0, respectively; 11.1%, 61.1%, 27.8%, and 0% at T2; and 11.1%, 50.0%, 38.9%, and 0% at T3. In addition, the mean change of the total Healing Scale score was 5.3 (95% CI: −1.2 to 11.8, *p* = 0.106, ES = 0.39) at T2 and 6.6 (95% CI, 1.0–12.3, *p* = 0.024, ES = 0.49) at T3 (Table 2). Regarding subscales, Nagomi was significantly improved with a large ES at T2 and T3. Kiyoraka and Uruoi were significantly improved with moderate ES at T2 and T3, respectively.

**Table 2.** The Change of Healing Scale

Variables	T0		T2 (The change of scores from baseline)				T3 (The change of scores from baseline)			
	Mean	SD	Mean	95% CI	*p* value	ES	Mean	95% CI	*p* value	ES
Total Healing Scale score	20.9	13.5	5.3	(−1.2 ∼ 11.8)	0.106	0.39	6.6	(1 ∼ 12.3)	0.024^*^	0.49
Nagomi	3.7	2.4	1.8	(0.4 ∼ 3.3)	0.018^*^	0.75	1.7	(0.2 ∼ 3.1)	0.024^*^	0.67
Kiwami	3.2	2.5	0.1	(−0.8 ∼ 1)	0.805	0.04	0.6	(−0.5 ∼ 1.7)	0.255	0.24
Kiyoraka	3.9	2.5	1.3	(0.1 ∼ 2.6)	0.042^*^	0.56	1.2	(0.2 ∼ 2.3)	0.025^*^	0.52
Uruoi	4.1	2.7	1.3	(0.1 ∼ 2.6)	0.038^*^	0.52	1.6	(0.5 ∼ 2.6)	0.005^*^	0.59
Hazumi	2.4	2.2	0	(−1.1 ∼ 1.1)	1.000	0	0.6	(−0.4 ∼ 1.6)	0.207	0.32
Mushin	3.5	2.4	0.7	(−0.6 ∼ 1.9)	0.264	0.29	0.9	(−0.4 ∼ 2.3)	0.164^*^	0.38

95% CI, 95% confidence interval; ES, effect size.

^*^ *p* < 0.05.

### The change of symptoms

The change of symptoms is shown in Table 3. Anxiety was significantly improved by a mean score of −1.2 (95% CI: −1.99 to −0.34, *p* = 0.008, ES = 0.52) at T2 and −1.2 (95% CI: −1.99 to −0.34, *p* = 0.008, ES = 0.52) at T3. Tiredness and shortness of breath were significantly improved by a mean score of −0.6 (95% CI: −1.18 to −0.04, *p* = 0.037, ES = 0.35) at T2 and −1.0 (95% CI: −1.72 to −0.28, *p* = 0.010, ES = 0.42) at T3. Well-being was significantly improved by a mean score of −1.3 (95% CI: −2.58 to −0.09, *p* = 0.037, ES = 0.54) at T3. The physical distress score, psychological distress score, and symptom distress score were also improved.

**Table 3.** The Change of Symptoms

Variables	T0		T2 (The change of scores from baseline)				T3 (The change of scores from baseline)			
	Mean	SD	Mean	95% CI	*p* value	ES	Mean	95% CI	*p* value	ES
Pain	1.7	1.6	−0.3	(−0.75 ∼ 0.08)	0.111	−0.19	0.1	(−0.34 ∼ 0.45)	0.773	0.06
Tiredness	2.2	2.3	−0.6	(−1.18 ∼ −0.04)	0.037^*^	−0.35	−0.4	(−1.1 ∼ 0.32)	0.261	−0.17
Drowsiness	2.2	2.3	0.2	(−0.69 ∼ 1.13)	0.614	0.09	0.3	(−0.75 ∼ 1.41)	0.523	0.17
Nausea	0.3	0.6	−0.2	(−0.36 ∼ 0.02)	0.083	−0.17	−0.2	(−0.42 ∼ 0.09)	0.187	−0.17
Lack of appetite	2.8	3.3	−1.0	(−2.26 ∼ 0.26)	0.114	−0.30	−1.4	(−2.97 ∼ 0.08)	0.062	−0.46
Shortness of breath	2.3	2.4	−1.0	(−1.72 ∼ −0.28)	0.010^*^	−0.42	−0.8	(−1.79 ∼ 0.12)	0.083	−0.33
Depression	1.8	1.8	−0.8	(−1.72 ∼ 0.17)	0.100	−0.39	−0.7	(−1.54 ∼ 0.09)	0.079	−0.39
Anxiety	2.6	2.3	−1.2	(−1.99 ∼ −0.34)	0.008^*^	−0.52	−1.2	(−1.99 ∼ −0.34)	0.008^*^	−0.52
Well-being	3.8	2.4	−1.0	(−2.02 ∼ 0.02)	0.055	−0.42	−1.3	(−2.58 ∼ −0.09)	0.037^*^	−0.54
Physical distress score	11.6	9.3	−2.9	(−5.54 ∼ −0.24)	0.035^*^	−0.31	−2.4	(−5.53 ∼ 0.64)	0.113	−0.26
Psychological distress score	4.4	3.5	−1.9	(−3.51 ∼ −0.38)	0.018^*^	−0.57	−1.9	(−3.32 ∼ −0.45)	0.013^*^	−0.54
Symptom distress score	16	11.9	−4.8	(−8.7 ∼ −0.97)	0.017^*^	−0.40	−4.3	(−8.15 ∼ −0.51)	0.029^*^	−0.36

^∗^ *p* < 0.05.

### Patients’ global impression of change

A total of 66.7% of patients felt that their general conditions at T2 and T3 had improved.

### Sleep satisfaction

The proportions of patients satisfied with sleep the previous night were 44.4% at T0 and 66.7% at T4. In addition, the level of sleep satisfaction at T4 compared with T0 was improved in 50.0%, remained unchanged in 38.9%, and worsened in 11.1%. There was no change during this study to any participant medication that may affect sleep quality.

### Heart rate variability

The mean LF/HF was 1.79 (SD: 0.60) during T0-1, 1.90 (0.93) during T1-2, and 1.85 (1.17) during T2-3. A decrease of LF/HF was noted in 55.6% during T1-2 and 44.4% during T2-3.

### Adverse events

No severe adverse events were reported throughout the study period.

## Discussion

The present study showed that HNIH improved the healing state and symptoms in terminally ill cancer patients. In addition, more than half of patients felt improvement of general conditions and sleep satisfaction and had a decreased LF/HF.

The most important findings revealed that HNIH improved the healing state, especially in subscales, including Nagomi: Relieved healing, Kiyoraka: Pure healing, and Uruoi: Refreshing healing, among terminally ill cancer patients. Other subscales, including Kiwami: Self-developed healing, Hazumi: Merry healing, and Mushin: Selfless healing, were not changed. Previous music interventions showed effectiveness on anxiety, depression, mood, and QOL among cancer patients with a long-life prognosis.^3^ However, the effect on healing has not been evaluated because of the lack of an instrument to assess healing. The Healing Scale was a unique questionnaire, developed to measure the healing state that arises from impressions of external objects.^10^ Healing is a subjective feeling. Palliative care for terminally ill cancer patients aims to decrease distress and improve QOL through pharmacological and nonpharmacological approaches.^17^ In this context, the provision of healing and relaxation for those patients is important, even short term. In addition, improvement of the autonomic nerve function among half of the participants suggests a relaxation effect. Previous studies showed that music intervention by a professional music therapist decreased the heart rate and systemic blood pressure among cancer patients with a good performance status.^18^ However, the present study also showed that only HNIH improved the autonomic nerve function, even in terminally ill cancer patients with a poor performance status. Among healthy persons, high resolution may lead to peripheral vasodilation and an increased arterial blood flow rate through the promotion of parasympathetic nervous system activity among healthy persons.^6^ In another study, high-resolution audio with inaudible high-frequency components induced a relaxed state through increases of alpha and low-beta electrical encephalography among healthy persons.^7^ In addition, HNIH led to decreased oxy-Hb concentrations in the right prefrontal cortex and a decreased LF/HF among healthy persons.^19^ Therefore, these mechanisms might also be involved in terminally ill cancer patients with severe distress.

The second important finding was that HNIH improved tiredness, shortness of breath, anxiety, well-being, and satisfaction with sleep among terminally ill cancer patients. In addition, it is important that ESs for fatigue and shortness of breath were moderate in the present population. These findings might also be correlated with the physiological effect of HNIH and with the subtypes of healing, including “Nagomi” as relieved healing, “Kiyoraka” as pure healing, and “Uruoi” as refreshing healing.

Only one randomized controlled trial of music intervention showed improved fatigue among patients receiving chemotherapy and with a good physical condition,^20^ and others showed negative results.^21^ Although pharmacological treatment, including corticosteroid and methylphenidate, and nonpharmacological treatment have been shown to improve fatigue, fatigue is one of the intractable symptoms among cancer patients. Therefore, patients are eager to receive more effective treatment for fatigue.^22^

Even though the effect of music intervention on shortness of breath has remained unknown, a systematic review showed a decrease in the respiratory rate.^3^ This suggests the effect of music on shortness of breath. In clinical settings, morphine, corticosteroid, and midazolam were recommended for shortness of breath.^23^ However, shortness of breath is also an intractable symptom. Considering the available treatments for this symptom, especially in terminally ill cancer patients, the presence of adverse effects is an important factor when selecting treatment. Music intervention may be a safe and promising future option.

Improvements of anxiety and well-being were also shown in this study, as in previous studies.^24^ In addition, the present study showed that half of participants reported increased satisfaction with sleeping. This intervention might affect sleeping.

This study had several limitations. The first is the generalization. Present exploratory study had a relatively small sample size, which has resulted in wide 95% CIs for our results. This indicated a degree of uncertainty in our results. Future research with a large sample size is necessary to enhance the precision of the results. The second is the lack of a placebo arm, because the present study was a single-centered, single-arm open-label trial. Present study results might be affected by placebo effects. Therefore, the effectiveness of HNIH could not be concluded in the present study, and randomized controlled trial is needed. Moreover, due to the single-arm design, the specific contribution of inaudible high-frequency components could not be determined. However, the present results suggest that inaudible high-frequency components may have some contribution, particularly in terminally ill cancer patients with severe distress, and further studies are required to explore this possibility. Third, the Healing Scale is not a commonly used instrument in the world. The developing process was adequately completed in Japan.^10^ But this scale is only currently available as the Japanese version. The unique nature of this scale, especially its subscales, may help it to become widespread in the future. A translation and validation study for English and other languages is needed. Fourth, multiple tests were performed in the present study. However, the present study is an exploratory study to find potentiate outcomes from many factors to plan the next confirmatory study. Therefore, we set a significance as *p* < 0.05 in the present study. Fifth, the Healing Scale was not validated among cancer patients. This scale was only validated among healthy population. However, no other questionnaire exists for healing. Sixth, this study used extensive equipment setup that is not readily available. Seventh, sleep satisfaction was evaluated by original question because measurement burden should be minimized. Next study should use validated questionnaire.

## Conclusions

The present single-arm study showed that the HNIH improved the healing state, especially relieved healing, pure healing, and refreshing healing, and symptoms, including anxiety, tiredness, shortness of breath, and well-being, among terminally ill cancer patients. In addition, more than half of patients felt improvement of general conditions and sleep satisfaction and showed decreased LF/HF. Further study to explore the use of this intervention and involvement of inaudible high-frequency components is needed.
